# Targeting TGFβ Signaling to Address Fibrosis Using Antisense Oligonucleotides

**DOI:** 10.3390/biomedicines6030074

**Published:** 2018-06-25

**Authors:** James T. March, Golnoush Golshirazi, Viktorija Cernisova, Heidi Carr, Yee Leong, Ngoc Lu-Nguyen, Linda J. Popplewell

**Affiliations:** Centre for Gene and Cell Therapy, School of Biological Sciences, Royal Holloway-University of London, Egham, Surrey TW20 0EX, UK; James.March.2013@live.rhul.ac.uk (J.T.M.); Golnoush.Golshirazi.2014@live.rhul.ac.uk (G.G.); Viktorija.Cernisova.2013@live.rhul.ac.uk (V.C.); Heidi.Carr.2014@live.rhul.ac.uk (H.C.); Yee.Leong.2016@live.rhul.ac.uk (Y.L.); Ngoc.Lu-Nguyen@rhul.ac.uk (N.L.-N.)

**Keywords:** fibrosis, transforming growth factor beta, antisense oligonucleotide

## Abstract

Fibrosis results from the excessive accumulation of extracellular matrix in chronically injured tissue. The fibrotic process is governed by crosstalk between many signaling pathways. The search for an effective treatment is further complicated by the fact that there is a degree of tissue-specificity in the pathways involved, although the process is not completely understood for all tissues. A plethora of drugs have shown promise in pre-clinical models, which is not always borne out translationally in clinical trial. With the recent approvals of two antisense oligonucleotides for the treatment of the genetic diseases Duchenne muscular dystrophy and spinal muscular atrophy, we explore here the potential of antisense oligonucleotides to knockdown the expression of pro-fibrotic proteins. We give an overview of the generalized fibrotic process, concentrating on key players and highlight where antisense oligonucleotides have been used effectively in cellular and animal models of different fibrotic conditions. Consideration is given to the advantages antisense oligonucleotides would have as an anti-fibrotic therapy alongside factors that would need to be addressed to improve efficacy. A prospective outlook for the development of antisense oligonucleotides to target fibrosis is outlined.

## 1. Introduction

**Figure 1:**
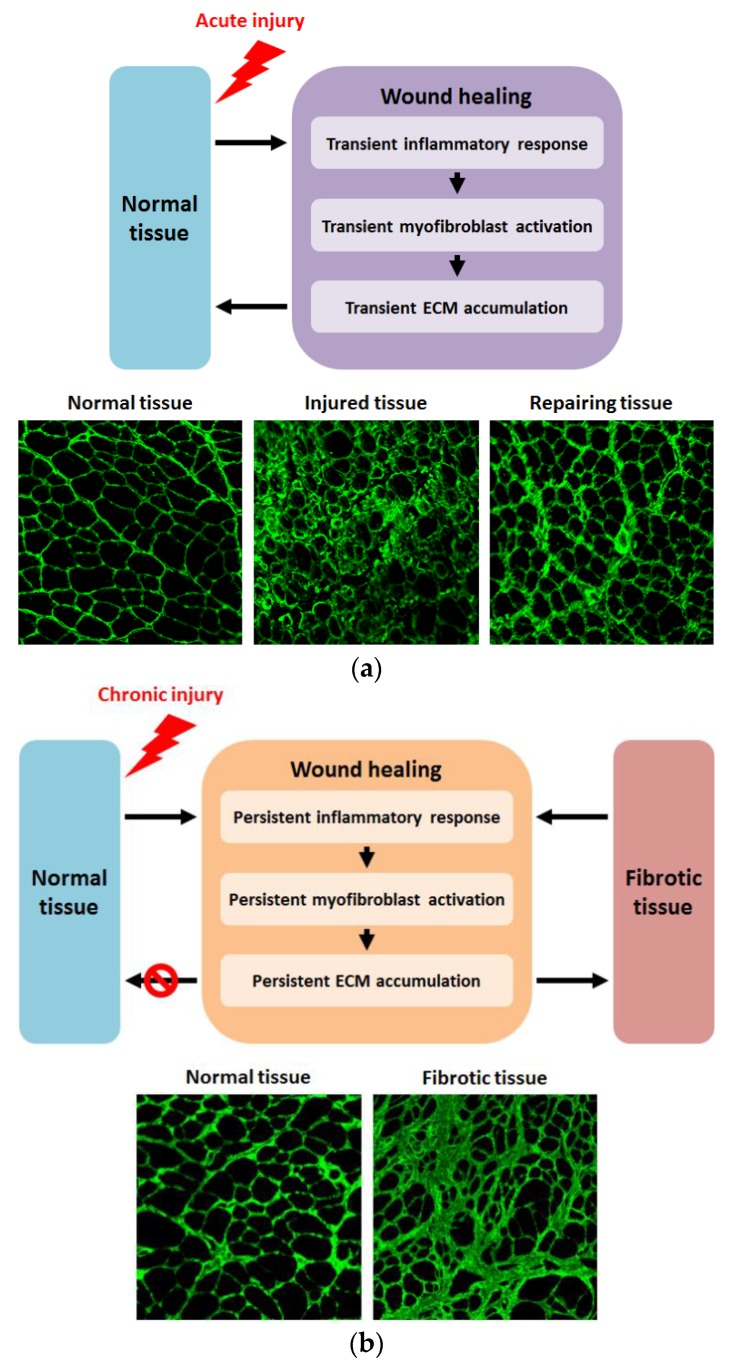
Outlines of normal wound healing following acute injury and fibrogenesis as a result of chronic injury: (**a**) Acute injury to normal tissue results in transient inflammation, leading to activation and proliferation of myofibroblasts, resulting in temporary ECM material accumulation, with restoration of ECM homeostasis once repair is complete and, ultimately, normalization of tissue architecture. Lower panels show cross-sections of normal (**left**), injured (**center**), and repairing (**right**) tibialis anterior muscle of 24-month-old wild-type mice immunostained for collagen VI at 200× magnification. Centre panel shows injured muscle 2 days after injury induced by injection with barium chloride, while right panel shows tissue repair 14 days post-injection, with muscle architecture beginning to normalize; (**b**) Chronic injury results in persistent inflammation, leading to recurrent myofibroblast activation and proliferation, which results in persistent and excessive ECM deposition and the development of fibrotic tissue. Lower panels show cross-sections at 200× magnification of the diaphragm of 9-month-old wild-type (**left**) and dystrophic *mdx* (**right**) mice immunostained for collagen VI. Wild-type muscle shows normal tissue structure, while *mdx* muscle displays fibrotic architecture with excessive and disruptive ECM deposition.

When a tissue is injured acutely, the pathological response results in wound healing (reviewed in Kumar, 2018 [1]), as outlined in Figure 1a. Pro-inflammatory cytokines recruit immune cells to the site of injury to remove tissue debris which leads to transient inflammation. The inflammation response is acutely amplified by the release of chemokines, such as interleukin-13 and interleukin-4, and the release of cytokines, such as transforming growth factor beta 1 (TGFβ1) and platelet-derived growth factor (PDGF), by the recruited immune cells themselves. This in turn leads to the temporary activation and proliferation of myofibroblasts to repair the insult. The resultant laying down of extracellular matrix (ECM) is transitory, with homeostasis between synthesis and degradation being re-established once the wound is healed through the proliferation of epithelial/endothelial cells.

Tissue fibrosis is characterized by the excessive accumulation of ECM and can arise because of disease inducing chronic tissue injury, or alternatively because of abnormalities in any contributor of the normal wound healing process. In disease contexts, fibrosis contributes to the phenotype of the disorder, particularly in later stages (reviewed by Walgraven and Hinz 2018 [2]). Additionally, fibrosis can affect many different tissues, some more so than others, despite having some mechanisms in common [3,4,5]. Regardless of trigger, be it chronic injury due to disease or abnormal signaling, fibrosis develops due to unremitting activation of normal tissue repair mechanisms. Persistent inflammatory response leads to continued myofibroblast activation resulting in excessive ECM production and fibrotic remodeling of tissue architecture (reviewed by Murtha et al., 2017 [6]), as outlined in Figure 1b. Many therapeutics targeting multiple components of the fibrotic pathway are at various stages of development (reviewed by Li et al., 2017 [7]). One approach that has shown much preclinical promise is the use of antisense oligonucleotides (AOs) to suppress expression of pro-fibrotic factors. Several beneficial features of AOs make this strategy attractive, such as their non-immunogenicity, the transience of target knockdown, the potential for flexibility in dosage, as well as the recent approval of two antisense oligonucleotide drugs for the treatment of genetic disease [8]. On this basis, this review will focus on the use of AOs as anti-fibrotic agents.

## 2. Transforming Growth Factor β Signaling in Fibrosis

The TGFβ superfamily of cytokines includes three TGFβ isoforms (TGFβ1–3), activins, inhibins, bone morphogenetic proteins (BMPs), growth and differentiation factors (GDFs) such as myostatin (also known as GDF8), and anti-mullerian hormone (AMH) [9,10]. TGFβ1, the prototypical member of this superfamily, is viewed as a critical molecular factor that drives the formation of fibrosis accompanying many disease states [11,12]. Indeed, TGFβ1 is persistently overexpressed in many fibrotic disorders and is strongly implicated as a principal driver of pathological fibrotic remodeling of different organs, including the lung [13,14], liver [15,16], kidney [17], heart [18,19], and muscle [20,21,22].

Another relatively well-characterized member of the TGFβ superfamily is myostatin. Myostatin is produced exclusively in skeletal muscles and acts mainly as a negative regulator of muscle development. Mutation of myostatin in humans and multiple animal species results in a hypermuscular and low body fat phenotype [23,24,25,26]. Therefore, modulating myostatin signaling has become an attractive approach to treat muscle wasting associated with muscular dystrophy, cancer cachexia, sarcopenia, trauma and diabetes [27,28,29,30,31,32]. Interestingly, while studying the effect of myostatin knockdown on muscle mass and strength, many groups have observed a corresponding reduction of fibrosis. For instance, in the *mdx* mouse model of Duchenne muscular dystrophy (DMD) combined with myostatin knockout, Wagner et al., 2002 identified that the diaphragm muscle of these animals had significantly less fibrosis compared with *mdx/mstn^+^* littermates [33]. Furthermore, other groups confirmed fibrosis reduction in mice treated with anti-myostatin neutralizing antibodies and peptides [27,34]. Researchers at The Johns Hopkins University were the first group to identify that myostatin could not only negatively regulate the growth of myocytes, but also directly regulate skeletal muscle fibrosis [35]. However, while some evidence indicates that myostatin stimulates the formation of cardiac fibrosis, it is not currently known what role myostatin might play in the formation of fibrosis in other organs, such as liver, kidney, and lung [10].

Both TGFβ1 and myostatin are synthesized in precursor forms consisting of a signal peptide, an N-terminal latency associated peptide (LAP) and a C-terminal domain that forms the active ligand [12]. Sequential proteolytic cleavages in the endoplasmic reticulum remove initially the signal peptide, and then release the LAP, which then binds to homodimers of mature ligand and keeps them in a latent propeptide complex that cannot interact with their receptors [12,36,37]. Additionally, in the case of TGFβ1, the LAP facilitates binding of the latent propeptide complex to the latent TGFβ binding protein (LTBP), which further keeps TGFβ1 in an inactive state [12,38]. Such latent propeptide complexes can be cleaved in the ECM by proteases such as matrix metalloprotease (MMP) 2 and MMP9 (in the case of TGFβ1) [12,39] or BMP1/tolloid-like metalloproteases (in the case of myostatin) [40] to release active ligand, which can subsequently bind to cognate receptors.

**Figure 2:**
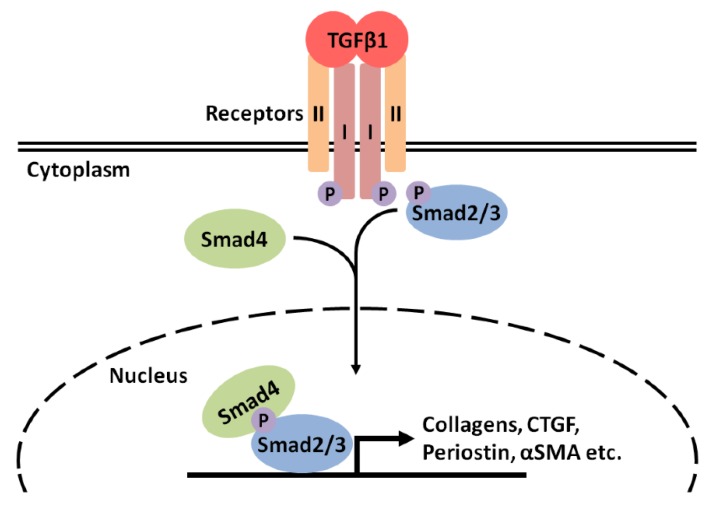
Outline of the canonical TGFβ1 signaling pathway. Upon activation, TGFβ1 binds to its cognate receptor, which consists of two type I (ALK5) and two type II (TGFβRII) subunits. Initial ligand binding is to TGFβRII induces the recruitment of ALK5. TGFβRII subunits phosphorylate ALK5 subunits initiating their activation. ALK5 subunits then phosphorylate the receptor-activated Smad proteins Smads 2 and 3. Phosphorylated Smad2/3 complex with Smad4 and then translocate to the nucleus to facilitate induction of expression of target genes, including profibrotic genes such as those for collagens, CTGF, periostin and αSMA. Canonical myostatin signaling proceeds in an analogous manner, but with different cognate receptors.

Although members of the TGFβ superfamily can signal through several pathways, the canonical pathway (outlined for TGFβ1 in Figure 2) involves the formation of a complex of activated ligand with a cognate tetrameric receptor, consisting of two type I (also known as activin-like kinase or ALK receptors) and two type II subunits with serine/threonine kinase activity [9,10,41]. A total of seven type I and five type II receptors have been identified, all forming complexes with different members of the TGFβ superfamily [10]. For example, TGFβ1 forms complexes with the type I receptor ALK5 and the type II receptor TGFβ receptor II (TGFβRII), while myostatin binds to ALK4 or ALK5 and the type II receptor activin receptor type IIB (ActRIIB) [9,10,41]. Upon ligand binding, the two types of receptor kinases act sequentially, with initial binding to the type II receptor inducing the recruitment and phosphorylation of type I receptors [9,10,41]. Upon phosphorylation, the type I receptors become active and phosphorylate receptor-activated Smad proteins, which in the case of TGFβ1 and myostatin are Smads 2 and 3 [9,10,41,42]. Upon phosphorylation, Smad2/3 forms heteromeric complexes with Smad4, which then translocate to the nucleus and facilitate activation of expression of target genes [9,41].

Many target genes induced by TGFβ1 via this signaling pathway are downstream pro-fibrotic genes, such as those encoding the ECM components fibronectin and different collagens [11,14,18,43,44,45,46], proteoglycans [47,48], plasminogen activator inhibitor-1 (PAI1) [49,50], integrins [51], connective tissue growth factor (CTGF) [52,53,54,55,56], periostin [57,58], tissue inhibitor of metalloproteases (TIMPs) [45], and α-smooth muscle actin (αSMA) [46,59,60]. Overall, the stimulation of pro-fibrotic genes by TGFβ1 promotes the differentiation of quiescent fibroblastic cell types to a pro-fibrotic, αSMA-positive, collagen-secreting, myofibroblastic phenotype in liver [46], lung [14], kidney [60], heart [18], and muscle [61]. Ultimately, this results in the fibrogenesis seen in many pathological conditions. Likewise, myostatin also stimulates expression of pro-fibrotic genes in this manner [10]. A pro-fibrotic environment in dystrophic skeletal muscle also appears to be promoted by myostatin through the induction of muscle fibroblast proliferation, as well as through the inhibition of fibroblast apoptosis [62]. The resulting increase in fibroblast number stimulates ECM accumulation and fibrotic alterations to muscle architecture [62].

Additionally, certain factors appear to modulate TGFβ signaling. For instance, it has been suggested that connexin43 contributes to the TGFβ1-mediated induction of αSMA transcriptional activity [63]. Connexin isoforms, such as connexin43, oligomerize to form connexins, hemichannels comprised of six connexin subunits, which in turn dimerize to form gap junctions, transmembrane intercellular channels that allow direct signaling between cells and the exchange of small (<1 kDa) metabolites and ions [64,65]. Connexin43 has been extensively studied and is found in many tissues including eyes, oral tissue, skin, and heart, and evidently contributes to the fibrosis seen in these tissues [63,66,67,68]. In part, this contribution of connexin43 to fibrogenesis appears to proceed via the TGFβ1-mediated activation of fibroblasts to myofibroblasts via αSMA induction [63,68]. Connexin43 may contribute to this induction of αSMA by the release of Smad2/3 from anchors on microtubules via competitive binding to microtubule anchor-points [69]. This would lead to increased availability of Smad2/3 for phosphorylation by the TGFβ1 receptor complex and thus increased activation of target genes, such as αSMA [69].

Some micro RNAs (miRs) also seem to exert regulatory effects on TGFβ signaling, and in so doing act as both suppressors and inducers of fibrogenesis. For example, studies have indicated that miR-29 functions as a negative regulator of TGFβ induced fibrosis, while also being downregulated itself through TGFβ signaling mechanisms in certain fibrotic contexts [70,71,72,73,74]. However, other miRs, such as miR-21 and miR-192, act to promote fibrosis through induction of TGFβ1 expression [75], or through inhibition of negative regulation of TGFβ1 signaling to amplify the pro-fibrotic signal [76,77].

## 3. The Use of AOs to Target TGFβ Signaling to Inhibit Fibrosis

On account of the evident critical role of TGFβ signaling in fibrosis, TGFβ1 and myostatin represent potential targets for functional antagonism or downregulation of expression, to prevent proliferation of pathological fibrotic tissue. TGFβ1 has been targeted with neutralizing anti-TGFβ1 monoclonal antibodies [78,79,80], TGFβ1-targeting inhibitor peptides [81], and soluble TGFβRII as a TGFβ1-ligand trap [82]; while myostatin has also been targeted with neutralizing anti-myostatin antibodies [27,83], myostatin propeptides [34,84], myostatin antagonists [85,86,87], and soluble myostatin receptors [88]. In addition to targeting these factors themselves, strategies have been utilized to suppress TGFβ signaling via targeting the cognate receptor complexes or SMAD2/3 for the inhibition of fibrogenesis. For example, small molecule inhibitors have been used to repress the function of TGFβRI/ALK5 and TGFβRII [89,90,91]. Besides these strategies, AOs themselves have been utilized to target TGFβ signaling at multiple points, including components of the TGFβ signaling cascade, such as ALK5 and SMAD3, and modulators of TGFβ signaling, such as connexin43 and miRs, for the amelioration of pathological fibrogenesis.

### 3.1. TGFβ1

In the case of TGFβ1 itself, AOs have been utilized to repress expression in several fibrotic models. For example, in vivo treatment with AOs targeting the translation initiation region of the TGFβ1 mRNA resulted in reduced TGFβ1 expression and suppression of ECM expansion in the anti-Thy 1.1 antibody model of glomerulonephritis and the unilateral ureteral obstruction (UUO) model of tubulointerstitial fibrosis [92,93]. Likewise, treatment with similar TGFβ1-targeting AOs reduced expression of TGFβ1 and the fibrotic ECM components fibronectin and collagen IV in in vitro and in vivo mouse models of diabetic renal hypertrophy [94]. AOs targeting TGFβ1 have also been utilized to preclude scarring during flexor tendon repair in mice [95]. These AOs inhibited TGFβ1 expression, reduced collagen III expression, and improved tendon repair by reducing the formation of fibrous adhesions between the tendons and the synovial sheath [95]. An alternative approach has utilized viral delivery of an antisense-RNA complementary to the 3′ coding sequence of the TGFβ1 mRNA to reduce TGFβ1 expression, signaling, and target gene expression, such as expression of αSMA and collagen I, by activated hepatic stellate cells [96].

### 3.2. Myostatin

AOs have been utilized to disrupt the myostatin pre-mRNA by inducing disruptive exon skipping to suppress myostatin expression. The efficacy of utilizing AOs for myostatin knockdown has been demonstrated in *mdx* mice [97,98,99]. Of note, we and others experienced that antisense treatment was significantly more effective when the AOs were administered locally into the muscle [97], rather than systemically by intraperitoneal or intravenous delivery [98,99]. Insufficient uptake into target tissues, high accumulation in the liver, and rapid clearance via the kidney are potential reasons for this limitation [100]. In particular, skeletal muscle-derived myostatin undergoes complex post-translational modification to maintain the balance of mature/precursor protein in the blood. Once the balance is disrupted, i.e., by exogenous compounds, precursor myostatin in its latent form can be activated to regain the balance, diminishing the therapeutic benefit [101]. These obviously are major drawbacks in the antisense approach for myostatin knockdown, compared to other strategies suppressing myostatin at protein level, and should be considered in any experimental plan. However, such problems can be overcome by using higher AO doses, or more frequently repeated AO delivery; feasible approaches given that AOs can be manufactured in large scale in a standardized manner.

### 3.3. ALK5

The ALK5 receptor (TGFβRI) has been targeted with AOs for the inhibition of fibrosis ex vivo in cultures of Dupuytren’s patient tissue [91], and in the *mdx* mouse model of DMD [102]. In ex vivo Dupuytren’s cultures, ALK5-targeting AOs appeared to suppress aspects of the fibrotic phenotype through the suppression of TGFβ1 signaling [91]. Likewise, in multiple cell lines ALK5-targeting AOs also inhibited TGFβ1 signaling, while in *mdx* mice these AOs decreased the expression of pro-fibrotic collagen III, CTGF and PAI1 [102]. However, ALK5-targeting AOs did not reduce the extent of fibrosis in the mice compared to controls, an effect that may, as Kemaladewi et al., 2014 point out, be due to the relatively young age of the mice or the very limited length of treatment utilized in the study [103]. 

### 3.4. Smad3

AOs have also been used to target Smad3 for the suppression of TGFβ1 signaling. Expression of Smad3 appeared to be blocked following AO treatment in keloid fibroblasts, an effect which was accompanied by suppression of collagen I expression [104]. Additionally, targeting Smad3 with AOs ameliorated flexor tendon scarring and improved healing after injury and surgical repair in mice [95]. A further study utilized a dual function synthetic oligodeoxynucleotide combining antisense and decoy approaches to target both TGFβ1 and Smad3 [105]. This approach inhibited both translation of TGFβ1 mRNA and Smad3 binding to promotors and was utilized to downregulate TGFβ1 expression and TGFβ1-dependant gene expression [105]. This resulted in repression of activation of hepatic stellate cells to a αSMA-positive myofibroblast phenotype, as well as suppression of fibronectin and collagen I expression, deposition of collagen fibrils, and attenuation of fibrogenesis in vivo in the CCl_4_-treated model of liver cirrhosis [105].

### 3.5. Connexin43

Connexin43 expression has also been suppressed using AOs, leading to reduced scarring in the eyes and skin after damage [66,68]. In the case of partial thickness burn wounds, this effect appears to be mediated by suppressing the inflammatory response, with reduced neutrophil numbers at early time points after injury [66]. This appears to facilitate tissue healing and prevent fibrosis as the proliferation of epidermal cells was enhanced and the re-epithelialization of the wound was accelerated in treated tissue compared to controls [66]. AOs have also been utilized to knockdown expression of endogenous connexin43 to demonstrate the TGFβ1-mediated induction of αSMA transcriptional activity in vivo [63]. Additionally, AO-mediated knockdown of connexin43 after glaucoma trabeculectomy resulted in decreased αSMA expression, reduced myofibroblast activity, as well as less densely packed collagen compared to controls [68].

### 3.6. miRs

Anti-miR or antagomir AOs have been used in several studies to target miRs in the hope of amelioration of fibrosis. For example, AOs have been utilized to target miR-21 in cardiac, kidney, and lung fibrosis [76,106,107]. Treatment with an anti-miR-21 AO resulted in downregulated expression of pro-fibrotic factors, such as collagens and other ECM constituents, in a pressure-overload-induced mouse model of cardiac fibrosis [106]. Similar results were seen in response to treatment with anti-miR-21 AOs in UUO- and ischemia/reperfusion injury-induced mouse models of renal fibrosis, with down regulation of collagens I and III observed [107], as well as in bleomycin-induced pulmonary fibrosis in mice, where upon AO treatment the pro-fibrogenic activity of TGFβ1 was reduced and deposition of ECM components, such as fibronectin and collagen, was suppressed [76]. Alongside these studies, other groups have used AOs to target other miRs for the suppression of fibrosis. In a mouse model of diabetic nephropathy, treatment with AOs targeting miR-192 decreased expression of TGFβ1, collagens, and fibronectin [75]. Anti-miR-208a AOs attenuated periarteriolar fibrosis in a model of rat heart failure [108]; while cardiac fibrosis was ameliorated in response to anti-miR-15 AO treatment in mice following ischemic injury [109]. Finally, AO-mediated inhibition of miR-34a appeared to reduce fibrosis following myocardial infarction in rats [110]; while targeting miR-34a, -b and -c likewise reduced cardiac fibrosis improved systolic function in mice subjected to pressure overload [111].

## 4. The Use of AOs to Target Expression of Downstream Fibrotic Mediators of TGFβ1

Additionally, other studies have sought to address fibrosis using AOs to downregulate expression of downstream pro-fibrotic effectors of TGFβ1, such as CTGF, periostin and TIMPs.

### 4.1. CTGF

Expression of the cytokine CTGF is undetectable in normal tissue but is promoted in wounded tissue before returning to basal levels following completion of tissue repair [112]. This upregulation of CTGF expression is correlated with the development of fibrosis in various disorders and models affecting different tissues including skin [113,114], liver [115], kidney [116], lung [53], and muscle [117]. This increase in CTGF expression appears to be induced by TGFβ1, and CTGF seems to function as a downstream fibrotic-mediator of TGFβ1 [52,53,54,55,56]. Indeed, it has been demonstrated that the induction of CTGF by TGFβ1 is mediated by a unique TGFβ1 response element within the CTGF gene promotor sequence [52]. 

CTGF therefore represents a potential target, downstream of TGFβ1, for the inhibition of fibrosis. A range of strategies have been investigated for suppression of CTGF expression or activity, including neutralizing anti-CTGF monoclonal antibodies [118,119,120,121] and CTGF-targeted small interfering RNAs [122,123,124]. Additionally, AOs have been utilized to block expression of endogenous CTGF for the suppression of fibrogenesis in multiple systems. For example, TGFβ1-induced expression of fibronectin and collagen I appears attenuated in vitro in rat renal fibroblasts via repression of CTGF expression using CTGF-targeting AOs [125,126]. Likewise, in vivo treatment with CTGF-targeting AOs reduced expression and interstitial deposition of ECM constituents, such as fibronectin and collagen I, reduced expression of α-SMA, and ameliorated development of renal fibrogenesis after UUO in rat and mouse models of diabetic nephropathy [127,128]. Furthermore, CTGF-targeted AOs also abrogated hypertrophic scarring in rabbit wound models [129], and inhibited collagen III expression, ameliorated fibrosis, and improved function during murine flexor tendon repair [95].

### 4.2. Periostin

The matricellular protein periostin (also known as osteoblast-specific factor 2), which was first identified in a screen of a mouse osteoblastic library, is normally expressed in the periodontal ligament and the periosteum, where it functions to modulate cell-ECM interactions and cellular response to external stimuli [130,131,132,133,134]. Periostin evidently serves in the normal tissue repair process across the body [57,135,136,137], but is also associated with fibrosis in multiple pathological conditions affecting different organs, including the heart [138,139,140,141], lung [58,142,143], kidney [17], skin [57], and muscle [137]. 

Within the ECM, periostin interacts with collagens I and V, fibronectin and tenascin C [142,144,145]. Additionally, periostin can stimulate collagen fibrillogenesis and crosslinking through interactions with BMP1, which promotes the proteolytic activation of lysyl oxidase, the enzyme responsible for collagen crosslinking [140,143,144,145,146]. Furthermore, periostin aids cell motility by serving as a ligand for integrins, such as αvβ3 and αvβ5 [140,147,148,149,150]. During acute injury, expression of periostin is temporarily induced by TGFβ1 (as well as other factors such as interleukin-4 and interleukin-3) [57,58,142]. After induction, periostin stimulates migration of cells such as myofibroblasts, while also serving to create a scaffold, using fibronectin, for the crosslinking of type I collagen, which engenders remodeling of ECM architecture and contributes to tissue repair. However, during chronic injury, due to persistent damage or stress, levels of periostin remain elevated, which contributes to the development of fibrosis in this context.

Since upregulated periostin expression is evidently associated with fibrogenesis, this pro-fibrotic factor is an attractive anti-fibrotic target downstream of TGFβ1. AOs were utilized to inhibit periostin in an *N*^G^-nitro-l-arginine methyl ester hydrochloride (l-NAME)-induced rat model of chronic kidney disease (CKD), with treated rats showing reduce fibrosis and tubular dilation [17]. Furthermore, a later study showed that diabetic mice treated with periostin-binding DNA aptamers displayed lower levels of renal fibrosis arising from diabetic nephrology [151]. Specifically, the DNA aptamers abrogated significant increases in fibronectin and collagen I mRNA and protein levels, while tubulointerstitial fibrosis was significantly reduced in treated mice [151]. In another mouse model of fibrosis, namely adhesion formation following abdominal surgery, treatment with periostin-targeting AOs significantly reduced adhesion formation 14 days after surgery, as well as significantly reducing TGFβ1 and collagen I levels, compared to saline or negative sense oligonucleotide treated mice [152]. Likewise, bleomycin-induced pulmonary fibrosis was effectively suppressed using intrathecally administered AOs targeting periostin, a treatment which also inhibited the metastatic colonization of the lung by melanoma cells [153]. The role of periostin in metastasis is well established (reviewed by Ratajczak-Wielgomas and Dziegiel, 2015) [154]. In line with this, inhibition of periostin using promoter-directed small antisense non-coding RNAs in vitro prevented the exponential expansion and motility of tumor cells [155].

### 4.3. TIMPs

MMPs are a large group of proteinases that are capable of degrading various kinds of ECM components, and which regulate ECM remodeling [156,157]. In healthy tissue, during wound healing, and in fibrotic tissues, the activity of MMPs is determined by the amount of active protein and the concentration of TIMPs, specific MMP inhibitors [157]. TIMPs are also involved in various biological processes such as cell survival, apoptosis, cell invasion, cell migration, cell growth and differentiation, and angiogenesis [157]. The TIMP family members include TIMP1-4, with TIMP1 and TIMP2 serving to protect components of the ECM from proteolysis through inhibition of MMP9 and MMP2 respectively [158,159]. TIMPs also function in their own right to enhance collagen synthesis [157]. Given their apparent role in regulation of ECM components and architecture, TIMPs represent potential targets for anti-fibrotic approaches.

For example, an antisense TIMP1 cDNA delivered using retroviral vector via intratracheal injection has been reported to suppress the early stages of fibrosis in the bleomycin-induced rat model of pulmonary fibrosis [160]. Up to three days post-bleomycin treatment, TIMP1 expression and hydroxyproline content were significantly decreased in lung tissue compared to controls [160]. Additionally, in rat models of hepatic fibrosis, AOs have been successfully used to suppress expression of both TIMP1 and TIMP2 [161,162]. It should be noted that on histological examination and by serum liver function index measurement, AOs targeting TIMP2 not only reduced hepatic fibrosis but also acted to improve liver function [162]. This may be due to its specific inhibition of MMP2 which is important in the synthesis and degradation of collagen IV in liver fibrosis [162].

## 5. Future Perspectives

AOs have been exploited pre-clinically to target several pro-fibrotic genes with in vitro and in vivo demonstrations of efficacy (see Table 1 for a summary of this).

Several features make the antisense approach an attractive strategy to pursue further. These advantages include flexibility in dosage, frequency of dosing, non-immunogenicity upon repeat administration, and transient effect. Perhaps most importantly, they could hold a possible preferential path to clinics since AOs for the treatment of genetic diseases, such as DMD and spinal muscular atrophy, are either in trial or have even approved [8]. One particular benefit of the use of AOs is that the transient nature of the target knockdown affords ease of dose manipulation. This would allow the removal of target inhibition when required, such as during times of injury, surgery, or other assault to non-fibrotic tissues, to ensure efficient repair can proceed. In particular, this may be necessary when using anti-fibrotic AOs in contexts when fibrosis is an accompanying pathological feature of disease, but not the direct cause, as is the case in some inherited disorders such as DMD. In these circumstances, the inhibition of pro-fibrotic mediators may interfere with or delay normal wound healing upon injury to tissues unaffected by the disorder. To allow tissue repair to proceed it would be expedient to suspend treatment, and the AO approach would easily allow for this possibility. Overall, the advantages of AOs make the antisense approach attractive compared to the limitations of other approaches, such as immunological blockade. For example, while AOs are non-immunogenic, antibodies are potentially immunogenic upon repeated treatment, although this can be minimized through use of humanized antibodies.

Additionally, in contrast to AOs being developed to treat certain genetic diseases, it is possible that with the right target, one AO could have a general application to fibrosis across a range of tissues, potentially leading to more rapid approval clinically for the treatment of a number fibrotic disorders. However, it is acknowledged that there is some diversity in the fibrotic pathways in different tissues, and so it is unlikely that one AO will treat all fibrotic disorders. Furthermore, due to the variety of pro-fibrotic pathways, it is likely that fibrosis in different tissues will respond differently to knockdown of the same target because of the interplay of other pathways specific to each tissue type. As such, it is likely that for effective targeting of fibrosis in any one tissue, more than one pro-fibrotic protein will need to be inhibited simultaneously. It should be noted that these issues would hold irrespective of the anti-fibrotic targeting strategy developed, be it AO, antibody, small molecule inhibitor or another approach. Nevertheless, it may be the case that the different knockdown strategies will behave differently in different tissues due to variations in tissue-specific fibrotic mechanisms. As such, it may be necessary to utilize a mixture of knockdown strategies for different targets in any one tissue. Finally, it would be of immense benefit and interest if a direct comparison of the different strategies available for knockdown for each target in each tissue was undertaken.

Other modes of targeting fibrosis, for example, using monoclonal antibodies, small chemicals, and enzyme inhibitors, have reached clinical trial with varying success [7]. This variability is largely attributable to the target addressed. For example, in a phase I/II trial, systemic sclerosis patients treated with a neutralizing anti-TGFβ1 antibody showed a significant increase in serious adverse events compared to those receiving placebo, while another trial discontinued early due to lack of efficacy [79,80]. Similarly, a clinical trial for DMD using a soluble myostatin receptor also reported adverse events and was eventually terminated due to potential safety concerns of epistaxis and telangiectasia [170]. In contrast, in trials, immunological blockade of CTGF proved to be more successful [118,121]. The same is likely to be true for AO-mediated knockdown of expression. A potential key conceptual advantage of targeting TGFβ1, or components of the TGFβ signaling cascade such as ALK5 or Smad5, is that this would suppress expression of a wide-range of downstream pro-fibrotic genes at the same time. However, TGFβ1 functions as a pleiotropic factor [171]. This potentially may result in adverse effects and is likely the reason for the events observed in the anti-TGFβ1 antibody trials [79,80]. It is probable that similar events would occur upon targeting components of the TGFβ signaling cascade as these factors are also involved in other biological processes in addition to fibrosis. A further source of concern is that mice in which the TGFβ1 gene is disrupted develop a wasting syndrome involving tissue necrosis, a multifocal inflammatory response in various organs, and eventual death [172]. This suggests that TGFβ1 does not represent a suitable target for anti-fibrotic therapeutic interventions. Additionally, some other pro-fibrotic targets summarized in Table 1, such as Kras and NF-κB, are involved in a range of cellular functions in addition to fibrosis. As such, these are also less attractive targets as their inhibition may affect other processes as well as fibrosis. In contrast, downstream pro-fibrotic mediators of TGFβ1, such as CTGF and periostin, could represent better targets due to their narrower range of functions. Thus, it is the opinion of the authors that the choice of target for any successful AO-mediated anti-fibrotic approach is critical, and that downstream mediators of TGFβ1 signaling, such as CTGF, are likely to be better targets than TGFβ1 itself.

Despite the apparent advantages and promise of AOs as anti-fibrotics, it must be acknowledged that to this point none of the anti-fibrotic AOs used in pre-clinical studies have reached clinical trial, despite the evident success of antisense therapy for inherited disorders. This disparity is likely to be due to the need for greater anti-fibrotic effects to be demonstrated in disease models than has been achieved to date. It is also evident that several different AOs targeting the same pro-fibrotic factor have been reported. A more concerted effort between groups to ensure that the optimal AO is trialed more comprehensively pre-clinically may accelerate the route to clinical trial.

Additionally, several drawbacks of the antisense strategy are evident. For instance, the potential benefits may be offset by limits to efficacy and associated toxicities of AOs [173,174]. Indeed, for fibrosis-targeting AOs to reach the clinic, enhancing efficacy and minimizing toxicity through optimization of targeted AO chemistry and delivery needs to be addressed. Nevertheless, much effort has been made to increase efficacy and mitigate potential toxicities, particularly through attempts to increase cellular uptake. Approaches, including conjugation of AOs to aptamers, cell-penetrating peptides, and antibodies, are currently being explored for those targeting disease causing mutations, such as those seen in DMD [97,98,175]. However, while myostatin-targeting AOs have been delivered using a peptide-conjugate delivery system in neonatal and adult *mdx* mice [97,98], to the knowledge of the authors, these strategies have not to this point been used to increase cellular uptake of AOs targeting other components of the TGFβ signaling pathway. Nevertheless, such approaches should easily be applied to AOs showing greatest efficacy against fibrosis. For instance, the development of cell-penetrating peptides in the past decade has successfully enhanced specific delivery of AOs in preclinical studies and holds promise to improve anti-fibrotic antisense therapy in the future in terms of improved efficacy and reduced toxicity [97,176,177,178,179].

Additionally, in many fibrotic conditions, addressing the fibrotic component of the disorder will not necessarily be corrective for the underlying disease etiology. For instance, in the context of DMD, targeting fibrosis will not correct the essential genetic defect, but merely mitigate some of the resulting effects. As such, combinatorial strategies should be developed that address both the fibrotic element of a disorder as well as the basic cause. Such an antisense approach has already been investigated with regards to recovering dystrophin expression and for myostatin knockdown to increase muscle mass as a potential treatment for DMD [97,98]. Furthermore, it may be pertinent to extend combinatorial strategies to cover other aspects of disease pathology. For example, there could be some benefit to use AOs to target inflammatory factors, particularly due to the role of inflammation in initiating the fibrotic cascade (ass Figure 1a). However, given the critical biological role of such factors, care should be taken.

Finally, as with most other therapies, an antisense approach would not reverse fibrosis, and would merely prevent further development of pathological fibrotic alterations to tissue architecture. Therefore, it is likely that prophylactic AO treatment would ideally be commenced upon diagnosis of a disease that has fibrosis as a pathophysiological component. For example, in the muscular dystrophies the genetic diagnosis is generally made before the onset of muscle fibrosis, while in diabetic kidney disease and fatty liver disease, fibrosis is also seen in the latter stages of these conditions. Early anti-fibrotic treatment is also critical because as fibrosis becomes established, deposited ECM may prevent cellular uptake of the therapeutic agent used to treat a disease. As such, an anti-fibrotic AO may only be effective at pre- and early-fibrotic stages. It is hoped that early treatment with AOs, or indeed any other therapy targeting pro-fibrotic signaling, would prevent the life-threatening consequences of the development of fibrosis in many different conditions, and should be further explored to improve treatment outcomes, enhance the quality of life of patients, and prolong life expectancy. In terms of healthcare, it will mean relief of the immense pressure for liver, kidney, heart, and lung transplantations, with associated savings in costs for treatments. We would therefore advocate that AO-targeted inhibition of pro-fibrotic protein expression holds realistic potential but would recommend that international cross-disease collaboration will be vital to achieve this aim.

## Figures and Tables

**Table 1:** Summary of profibrotic factors that have been targeted with AOs.

Pro-Fibrotic Factor	Function	Model	References
TGFβ1	Pro-fibrotic master—regulator	In vitro and in vivo rodent models of renal fibrosis	[92,93,94]
		In vivo mouse model of tendon scarring	[95]
		In vitro models of hepatic fibrosis	[96]
ALK5	Component of canonical TGFβ1 receptor—TGFβ1 signaling	Ex vivo in cultures of Dupuytren’s patient tissue	[91]
		In vivo mouse model of DMD	[102]
SMAD3	Component of canonical TGFβ1 signaling pathway	In vitro culture of human keloid fibroblasts	[104]
		In vivo mouse model of tendon scarring	[95]
		In vitro and in vivo mouse models of renal fibrosis	[105]
Connexin43	Component of gap junctions—pro-fibrotic factor	In vitro culture of rat cardiac fibroblasts	[63]
		In vivo model of rabbit eye glaucoma trabeculectomy	[68]
		In vivo neonatal mouse model of burn injury	[66]
miR-21	Pro-fibrotic miR	In vivo mouse model of cardiac fibrosis	[106]
		In vivo mouse models of renal fibrosis	[107]
		In vivo mouse models of pulmonary fibrosis	[76]
miR-192	Pro-fibrotic miR	In vivo mouse models of renal fibrosis	[75]
miR-208a	Pro-fibrotic miR	In vivo rodent models of vascular and cardiac fibrosis	[108,109]
miR-34	Pro-fibrotic miR	In vivo mouse and rat models of cardiac fibrosis	[110,111]
CTGF	Downstream pro-fibrotic effector of TGFβ1	In vivo neonatal mouse model of burn injury	[66]
		In vivo rabbit model of hypertrophic scarring	[129]
		In vivo mouse model of tendon scarring	[95]
Periostin	Downstream pro-fibrotic effector of TGFβ1	In vivo rat model of renal injury	[17]
		In vivo mouse model of renal fibrosis	[151]
		In vivo mouse model of surgically induced adhesions	[152]
		In vivo mouse model of pulmonary fibrosis	[153]
		Cultures of multiple human cell lines	[155]
TIMPs	MMP inhibitors and pro-fibrotic factors	In vivo rat model of pulmonary fibrosis	[160]
		In vivo rat model of hepatic fibrosis	[161,162]
bFGF *	Cytokine—pro-fibrotic factor	In vivo rat model of pulmonary fibrosis	[163]
Kras *	Monomeric GTPase—component of signal transduction pathways	In vivo rat model of renal fibrosis	[164]
Sp1 *	Transcription factor	In vitro culture of human dermal fibroblasts and in vivo in murine skin	[165]
NF-κB *	Transcription factor	In vivo mouse model of pulmonary fibrosis	[166]
		In vivo mouse model of interstitial fibrosis	[167]
STAT1 *	Transcription factor	In vivo rat model of pulmonary fibrosis	[168]
HSP27 *	Chaperone	In vitro and in vivo rat model of pulmonary fibrosis	[169]

* These factors are not discussed in the main text of the review.
